# Thermocapillary Marangoni Flows in Azopolymers

**DOI:** 10.3390/ma13112464

**Published:** 2020-05-28

**Authors:** Andrzej Miniewicz, Anna Sobolewska, Wojciech Piotrowski, Pawel Karpinski, Stanislaw Bartkiewicz, Ewa Schab-Balcerzak

**Affiliations:** 1 Advanced Materials Engineering and Modelling Group, Faculty of Chemistry, Wroclaw University of Science and Technology, Wybrzeze Wyspianskiego 27, 50-370 Wroclaw, Poland; anna.sobolewska@pwr.edu.pl (A.S.); w.piotrowski@intibs.pl (W.P.); pawel.karpinski@pwr.edu.pl (P.K.); stanislaw.bartkiewicz@pwr.edu.pl (S.B.); 2 Centre of Polymer and Carbon Materials, Polish Academy of Sciences, M. Curie-Sklodowska 34, 41-819 Zabrze, Poland; ebalcerzak@cmpw-pan.edu.pl; 3 Institute of Chemistry, University of Silesia, 9 Szkolna Str., 40-006 Katowice, Poland

**Keywords:** azobenzene derivatives, photochromic polymers, light-induced polymer mass movement, optofluidization process, relief formation, thermocapillary Marangoni effect

## Abstract

It is well known that light-induced multiple *trans-cis-trans* photoisomerizations of azobenzene derivatives attached to various matrices (polymeric, liquid crystalline polymers) result in polymer mass movement leading to generation of surface reliefs. The reliefs can be produced at small as well as at large light intensities. When linearly polarized light is used in the process, directional photo-induced molecular orientation of the azo molecules occurs, which leads to the generation of optical anisotropy in the system, providing that thermal effects are negligible. On the other hand, large reliefs are observed at relatively strong laser intensities when the optofluidization process is particularly effective. In this article, we describe the competitive thermocapillary Marangoni effect of polymer mass motion. We experimentally prove that the Marangoni effect occurs simultaneously with the optofluidization process. It destroys the orientation of the azopolymer molecules and results in cancelation of the photo-induced birefringence. Our experimental observations of polymer surface topography with atomic force microscopy are supported by suitable modelings.

## 1. Introduction

A growing demand for advanced materials and technologies for modern photonics, e.g., optical data storage, light processing, photomechanics and photofluidics, stimulates development of novel materials and tools for material modifications. Photochromic materials such as azo-functionalized polymers have been widely investigated [1,2,3,4,5] because of their unique features to undergo inducement of optical anisotropy and surface mass transport upon suitable laser light irradiation [6,7,8,9,10,11,12]. Recently, it has been demonstrated that the chiral symmetry of light (i.e., Laguerre-Gauss beam) with photons carrying orbital angular momentum can be mapped via mass transport and surface pattern formation in azopolymers [11], providing the polymer layer shows the optofluidization state. By applying a systematic approach to the in situ inscription by the holographic method of surface relief gratings (SRGs) under atomic force microscope (AFM), the mechanism of mass transport in azopolymer films has been clarified to a large extent [7]. However, the investigated azopolymers in different laboratories across the world differ from each other at the microscopic level both chemically and structurally. Therefore, their behavior under the same illumination conditions can vary depending on the specific material properties and chemistry (type of bonds, way of substitutions of different azobenzene derivatives, concentration of azobenzenes, polymer mass, polymer viscosity, glass transition temperature, etc.). Tremendous work has been devoted to the development of various groups of materials containing azobenzene derivatives in their structures. Azobenzenes have been investigated in various matrices including glasses [13], liquid crystalline polymers [14,15], biopolymers [16,17,18,19], liquid crystals [20,21,22,23], Langmuir-Blodgett-Kuhn structures [23], layer-by-layer films [24,25] monolayers [26,27] and even nanoparticles [28,29]. Particularly interesting and widely discussed is the phenomenon of light-induced macroscopic polymer mass transport discovered for azo-substituted polymers [30,31]. The transport is driven by the optical fields at temperatures well below the polymer glass transition temperatures (T_g_) and its magnitude and anisotropy largely depend on the used light polarization and light exposure (time and irradiance level). These features were carefully studied and explained for two-wave mixing (holographic) recording experiments using various types of beam polarizations (s-s, p-p, s-p, LCP-RCP and others) [1,2,3,4,5,6,7,8]. 

In this work we focused our attention on the less investigated [32,33,34,35,36], but still intriguing phenomenon of surface topography modification induced by irradiation of azopolymer layers with a focused linearly polarized single Gaussian laser beam. We wanted to check the process of optofluidization of the upper polymer layer under laser irradiation widely described in review papers by Lee et al. [4] and by Choi et al. [37], and search for the thermocapillary Marangoni effect [38] in systems studied by us. In all fluids the Marangoni effect is observed as the rapid near-surface flow of the fluid mass from the lower toward the higher surface tension area. In the experiment with the single beam the mass flow is expected from the focal point centrally outside it. We searched for this mass flow by changing the irradiation level and the observation of the surface reliefs using atomic force microscopy technique. The beam intensity was controlled and changed during the inscription process in order to investigate the switching mechanism between the electric field gradient driven and the light intensity thermally driven direction of the mass flow in azopolymers under weak and dense light illumination, respectively. The results are discussed in the framework of the molecular diffusion model formulated by Ambrosio et al. [11,39].

## 2. Materials and Methods 

**Figure 1:**
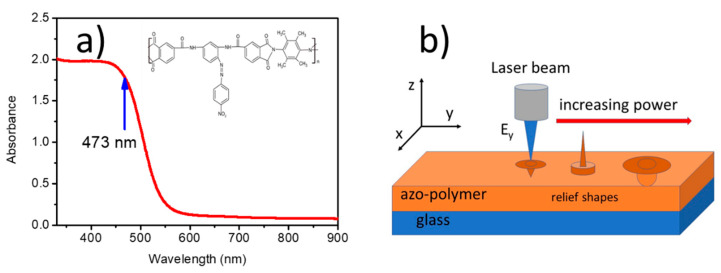
(**a**) UV-VIS absorption spectrum of the azo-functionalized poly(amide-imide) measured in 20-μm-thick film casted on the glass plate. Chemical structure of the polymer monomeric unit is pasted as the inset. (**b**) Scheme of an experimental setup used for single linearly polarized beam inscriptions. The three distinct surface deformation shapes after the action of the laser pulse, observed in our experiments, are schematically shown. The laser power increases from the left to the right. Deformations (not in scale in the figure) have amplitudes ranging from a few nanometers at weak power up to 800 nm.

For experiments on the microstructures inscription, we chose the azo-functionalized poly(amide-imide), for which the chemical structure and absorption spectrum are shown in Figure 1.

The choice of this type of polymer was conscious and dictated by the presence of the rigidly bound azo-chromophore pending groups to the polymer main chain. This type of azopolymer is not a liquid crystal polymer and the rigidly bound azo-chromophores facilitate the main chain movement coordinated with the multiple *trans-cis-trans* photoisomerizations induced by an external irradiation. The compound was prepared by the polycondensation reaction of diamidedianhydride (DADA) with 3,3’,5,5’-tetramethyl-benzidine, according to the procedure described in [40]. This polymer exhibits good solubility in common organic solvents due to the presence of the methyl groups and the bulky azo-chromophore group. The uniform and transparent films were easily cast from the solution of the polymer in DMA (dimethylanhydride), c = 10^−5^ mol/L on clean glass substrates. UV-VIS absorption spectrum of this compound measured for a 20-μm-thick film (cf. Figure 1a) exhibits a broad band centered at around 380 nm and extending up to 560 nm. The chemical structure of the azopolymer monomeric unit is shown in the inset of Figure 1a.

Inscriptions of microstructures in the azo-functionalized polymers have been done with the help of the inverted optical microscope (Olympus IX71 i, Olympus Europa Holding, GmbH, Hamburg, Germany). The microscope was fed with light coming from a quasi-cw single mode TEM_00_ blue laser (type MSL-FN-473). The maximum average power delivered by this laser was 100 mW. Its operation wavelength λ = 473 nm was absorbed by the azopolymer. The beam of the output diameter D ≈ 2 mm passed through the beam expander system, then through neutral density filters used for the intensity attenuation, the motorized half-wave plate for the azimuthal rotation of its polarization plane and a set of mirrors guiding it toward a microscope objective. The maximum power delivered directly to the sample lying on the microscope stage was 34 mW as measured by the Si-calibrated detector connected to the Field Master laser power meter. Using neutral density filters we were able to diminish the power incident on the sample to 30 μW. Working with the microscope objective 40× which produced a spot size of radius 1.6 μm, we get average light intensities in the range 0.4 kW/cm^2^ to 423 kW/cm^2^. Usually, we used microscope objectives of the magnification 40× as well as 100× without using any immersion liquid. For an objective 100× the maximum light intensity could reach nearly 4.3 MW/cm^2^. The sample was mounted on the nanopositioning x,y,z stage TRITOR 102 SG (Piezosystem jena GmbH, Jena, Germany) working in a closed loop motion with the maximum precision of ±2 nm and the maximum displacement range of 80 × 80 μm^2^. The homemade program allowed us to move the stage with a given x–y pattern and predesigned time sequence of the laser focus beam movement over the surface. The inscribed structures were in situ monitored using the Retiga 2000 CCD color camera (Qimaging Scientific Cameras, Teledyne Photometrics, Tucson, AZ, USA). Obtained reliefs and the surface topography were measured using the atomic force microscope. For the azopolymer surface scan we used the Veeco Dimension V AFM (Veeco Instruments Inc., Plainview, NY, USA) working in a tapping mode. Subsequently, results were elaborated with the WS × M 4.0 programming tool. Cross sections of the surface deformation were numerically modeled in the COMSOL Mulitiphysics using the theory developed by Ambrosio et al. [11,39] and Mathcad programing tools.

A schematic view of the polymeric layer inscribed by a focused laser beam under changing average laser power is shown in Figure 1b. In a real experiment the beam was coming from the bottom and a sample was inverted facing with the polymer layer toward the beam.

We employed different schemes of illumination, but always keeping a short time (50 ms) of laser irradiance at a single spot position. The spot-to-spot distance was set to 1 or 5 μm in order to avoid trace overlapping occurring at strong irradiances. During a series of inscriptions, the linear polarization of the laser beam was rotated between 0° and 90° directions. Occasionally, we also used an optical polarizing microscope to observe light-induced birefringence in the irradiated spots on the polymer surface.

## 3. Results and Discussion

### 3.1. Analysis of Direct Laser Inscriptions in an Azopolymer

We used the same polymer in the past for inscription of surface relief gratings [40]. SRGs were inscribed with the help of two-wave mixing method using 514.5 nm excitation light delivered from the cw Ar^+^ laser of 25 mW power (*I_average_* ≈ 350 mW/cm^2^). Under such conditions the recording time necessary to build up saturated gratings was on average about 30 min. Amplitudes of SRG determined by the atomic force microscopy were relatively weak, merely approaching 100 nm. However, both s-s (intensity modulation) and s-p (polarization modulation) gratings could be recorded in the investigated polymer [40] confirming its ability to exhibit mapping of local polarization variations within the grating period.

**Figure 2:**
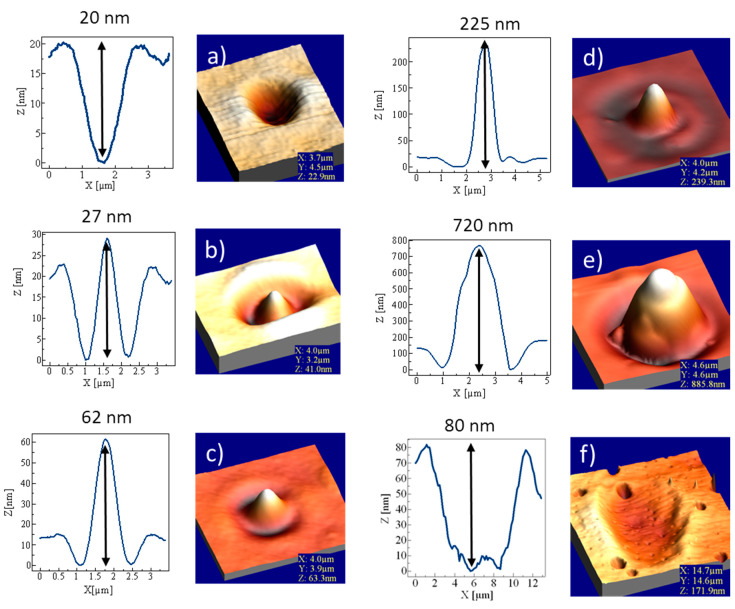
Relief structures observed by the atomic force microscope (AFM) surface topography in the azo-functionalized poly(amide-imide) generated by 50 ms irradiation with the linearly polarized focused Gaussian beam. Each 3D relief structure is accompanied on the left by a profile shape that has been measured at the central plane. Arrows are marked at the maximum relief amplitude. (**a**) Weak power regime (~50 μW)—no temperature effect; (**b**) first intermediate regime (~100 μW); (**c**) medium power regime (~130 μW)—inverted mass movement; (**d**) medium power regime (~500 μW)—toward maximum amplitude; (**e**) beginning of strong power regime (~1000 μW)—influence of temperature and optofluidization and (**f**) strong power regime (~3400 μW)—optofluidization and temperature-driven isotropic fluidization.

In this work, we study the reliefs under optical microscope and using a single beam focused to a spot. Here, we use much larger light intensities than those used for the holographic grating inscription, i.e., as high as *I_average_* ≈ 420 kW/cm^2^ (at λ = 473 nm and microscope objective 40×), but the exposition time was limited to 50 ms only. Under such dramatically different conditions, qualitatively different phenomena were observed. We observed three regimes of reliefs: (i) at weak laser powers (30–100 μW) shallow craters are formed with two side lobes (hills) situated along the linear polarization direction of the laser beam, which is the classically observed structure for which the induced birefringence is well visible [11,41]; (ii) at medium laser powers (100–1000 μW) the reverse effect takes place, i.e., at the beam focal point the mass of the polymer was strongly displaced above the surface level forming a relatively high cone-like structure surrounded by two rounded pits formed along the light polarization direction, with birefringence not seen in the beam center; and (iii) at strong laser powers (1000–34,000 μW) a shallow but much wider dip is formed surrounded by the rim, and no birefringence could be observed. In between these three regimes the two intermediate states appear clearly showing the rebuild of the reliefs. In Figure 2 we show the light intensity dependent relief structures across the three regimes of the inscription laser intensity mentioned above.

**Figure 3:**
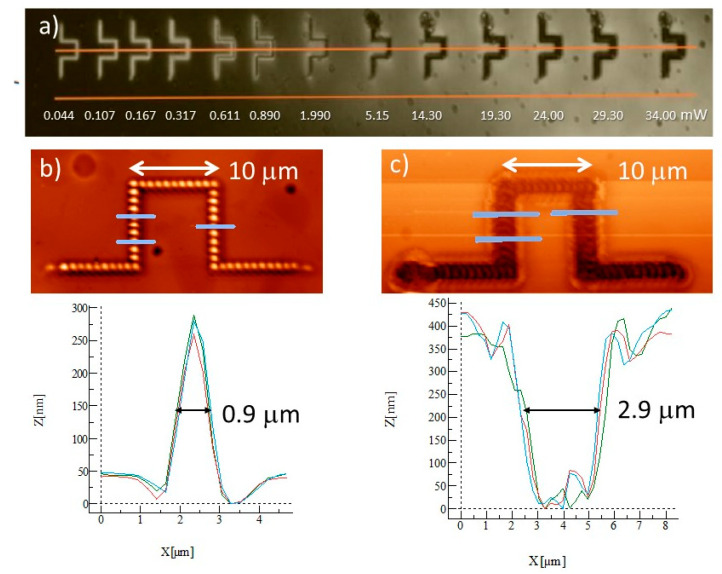
(**a**) Polarized optical microscopy (POM) photograph under crossed polarizers for the detection of photoinduced birefringence in the recorded structures for laser power increasing from 0.044 to 34 mW. Relief structures generated by 50 ms irradiation with the linearly polarized, focused by ×40 objective, Gaussian beam of (**b**) 0.61 mW and (**c**) 24 mW power as observed by the AFM surface topography. Below relief structures we present the plots of three profiles corresponding to places marked by blue lines.

At the small and medium power regime the affected area of the polymer is determined by the transverse size of the beam at the focus, i.e., the influenced area is comparable with the diameter of the gaussian beam, counted to the intensity value equal I_0_/e^2^, where I_0_ is the light intensity at the central point and e is Euler’s number. Smoothly, from the medium power regime toward the strong power regime, the polymer area influenced by the laser beam starts to widen significantly, evidencing that the thermally driven mass flow extends outside the beam diameter. In order to distinguish between the directional photofluidization described in Lee’s et al. work [4] and non-directional photofluidization processes we performed another experiment of direct laser inscription. We recorded the same structures (comprising of a chain, 30 μm in length and 10 μm in width, of points separated by 1 μm) in the function of increasing power. Next, we analyzed them with the polarized optical microscopy (POM) technique using Olympus BX60F5 microscope (Olympus Optical co. ltd. Tokyo, Japan) and AFM scanning. The results of the experiment are shown in Figure 3.

**Figure 4:**
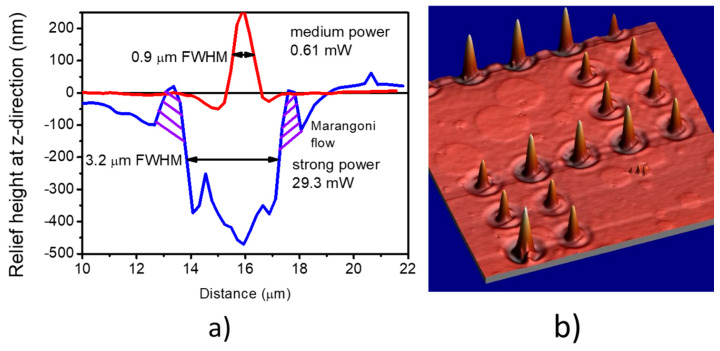
(**a**) Two profile shapes determined at the medium 0.61 mW (red line) and the strong 29.3 mW (blue line) power. Shadowed areas in the blue crater profile indicate features that arise outside the optical beam radius and that are, in our opinion, due to the optofluidization effect and the Marangoni-driven flow. (**b**) Topographical AFM study presenting a 3D view of surface deformation due to laser-induced point-by-point inscription within the medium power regime.

From Figure 3a, where the recorded structures were photographed under POM with partially crossed polarizers, one can see that at the left side of the figure the lines composed of 40 separate points look “white”. This clearly shows that the spots show light-induced birefringence. With the rise of the inscription laser power the central part of the line becomes “black”, showing lack of birefringence—the molecular disorder. At still higher powers, i.e., above 5.15 mW, lines are black and much wider that those observed at small powers (cf. Figure 3b,c with the linewidth change from being 0.9 to 2.9 μm). For laser powers not exceeding 0.6 mW, the lateral writing resolution, defined as the minimal distance between adjacently written features with no overlapping, is about 1 micrometer. In Figure 3c at both sides of the crater profile we can see two sharp anomalies which are characteristic for all reliefs produced with power above 5 mW; later on we will link these as arising from the Marangoni effect. In Figure 4a we show a plot comparing the relief profiles for the same polymer layer and the same illumination condition except the laser power marked in the figure. 

In Figure 4b we present an example of the inscription of the structures of points with lower density (they are now separated by 5 μm distance), with objective 100× and power of 0.66 mW. Reliefs similar to those shown in Figure 4b are rarely reported in the literature and classically they are dip-like structures with two lobes for the linear polarization and the rounded ring for the left or the right circular polarizations (cf. Figure 2 in Ref. [4], Figure 3 in Ref. [32] and Figure 3 in Ref. [41]). The reason is that most scientists are interested in molecular reorientation processes occurring at room temperature or at temperatures much below the glass transition temperature T_g_ of the studied polymers and consequently use small light intensities. At higher illumination levels the temperature of the polymer can locally rise above T_g_ and oriented photofluidization and then liquefaction processes take place. We must comment that the values of the laser power regimes characterizing qualitatively different surface reliefs described in this work are not general ones, but they are strictly related to the laser wavelength used, numerical aperture of the microscope objective and the material absorption. Polymer thermal properties and viscosity can also influence the extension and appearance of these regimes.

In the range below the T_g_ temperature of the polymer, the increase of temperature due to light absorption contributes to the rearrangement of the azo molecules due to the influence of the polarized pump light (*trans* isomers of azo-derivatives tend to align perpendicular to the polarization direction). When the local temperature of the polymer rises above T_g_, molecular chains begin to move, destroying the orientation and resulting in a drop of the photo-induced birefringence. At higher irradiance levels a liquified polymer layer behaves like a viscous liquid and its deformation is much easier and internal ordering ceases. Judging from the elongated cone-like relief shape at the beam center, accompanied with the lack of the birefringence just in the beam center, a different mechanism than the optical-field gradient force model [32] or the asymmetric diffusion model [42] for the mass transport must dominate within this regime.

### 3.2. Simulation of Reliefs within an Optofluidization Model

**Figure 5:**
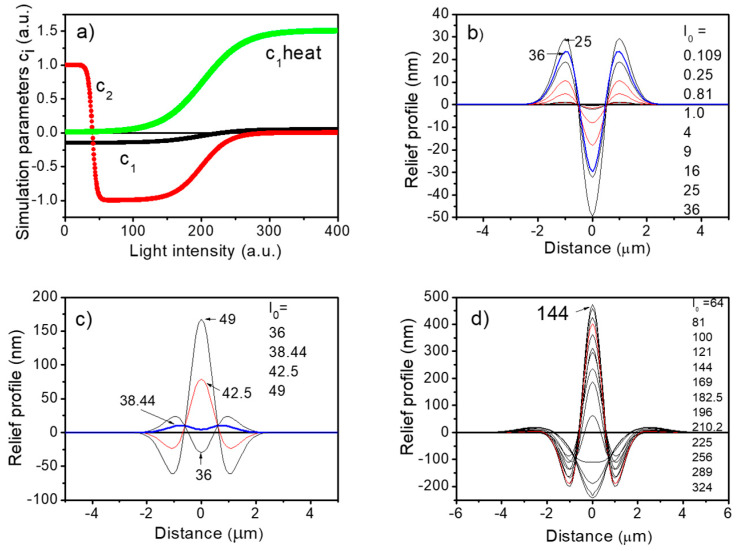
Results of the simulation of the laser-induced profiles in the azopolymer. Reliefs are extracted from three dimensional plots within the (y,z) plane, i.e., the light polarization plane. (**a**) Dependence of the simulation parameters (see Appendix A) in function of Gaussian beam intensity I_0_ expressed in arbitrary units. Simulation parameters are c_1_ = −0.15, c_2_ = 1.0 and c_3_ = 0. Additional c_1 heat_ parameter corresponds to the amount of heat released during irradiation and its spread. (**b**) Relief profiles in the weak power regime (I_0_ = 0 to 36 a.u.). (**c**) Relief profiles in the intermediate power regime (I_0_ = 36 to 49 a.u.). (**d**) Relief profiles in the medium (I_0_ = 64 to 196 a.u.) and the strong power regimes (I_0_ = 196 to 400 a.u.).

In order to numerically simulate all the experimentally measured reliefs in this work obtained under changing light irradiance level, one needs a general physical model of the light–matter interaction. Despite the richness of models published in the literature on the relief formation in azopolymers (cf. Table 1 in the review paper [4]) not one of them is able to properly predict all the features observed by us. The main difficulty lies in the molecular complexity of the so far investigated azopolymers, differing in their physicochemical properties and molecular structures and then responding differently to the same stimuli, e.g., intensity of the used light, its wavelength, polarization and pulse duration. We do not attempt to formulate any new model for the investigated poly(amido-imides), but we can show that by adapting the phenomenological model of the light-driven anisotropic diffusion concept [43,44,45,46], elaborated in papers by Ambrosio et al. [11,39], we may reproduce most of the results except the above-mentioned thermal effects of optofluidization and Marangoni flows that depend on surface tension changes due to temperature or concentration gradients. The strength of this approach lies in fact that the electromagnetic field of light impinging on the sample is treated rigorously in its full complexity, including various intensity distributions, polarization states and even beams carrying angular orbital momentum. Within this approach, the polymeric film is treated differently in its bulk and thin surface layer by introducing the enhanced molecular diffusion in the proximity of its free surface (i.e., allowing for an optofluidization phenomenon). The surface deformation Δh(0,y) resulting from the photo-induced polymer mass migration could be calculated from the simplified equation derived by Ambrosio et al. [39] and is given in the Appendix A. All the simulations were performed with Mathcad 2001 Professional programming tool. In order to properly simulate the polymer response Δh(0,y) to irradiation across the weak, medium and strong power regimes of the inscription, we created a functional dependence of the main parameters of the theory, namely c_1,_ c_2_ and c_3_ on the laser beam intensity I_0_ given here in arbitrary units (a.u.) (see Figure 5a).

The use of arbitrary units of intensity in simulations is the only rational choice as the azopolymer response depends on the amount of light absorption, the amplitude of the electric field of light and the amount of released heat.

This problem has not yet been addressed in the literature. In order to simulate the three different regimes of the power and relate to them totally different material responses, we have to propose the functional dependencies for the main parameters of the theory c_1_ and c_2_ in the function of Gaussian beam intensity I(x,y) at the maximum, i.e., I_0_ = I(0,0). As a starting point we took optimal parameters used in the reference [39] c_1_/c_2_ = −0.15, namely c_1_ = −0.15, c_2_ = 1.0 and c_3_ = 0, which perfectly matched our experimentally observed surface profiles in the weak power regime (here 0 to 36 a.u.). However, in entering the medium power regime (here 64 to 196 a.u.), sign inversion for the c_2_ parameter was necessary to qualitatively reproduce the formation of reliefs. The coefficient c2 is responsible for the mass flow in the direction parallel to the optical electric field. We decided to quite rapidly change this parameter to reproduce the changes in a real experiment. This was not necessary for the c_1_ parameter responsible for the mass flow driven by the gradient of the light intensity regardless of polarization. However, we have introduced an additional component proportional to the gradient of the light intensity c_1 heat_ that allowed us to properly reproduce the surface deformation in the strong power regime (here from 196 to 400 a.u.). The spread of heat described by the coefficient c_1 heat_ >> c_1_ and c_1 heat_ > 0 in the strong power regime adequately addresses what is observed experimentally, namely a crater-like feature surrounded by a rim. In the theory there is no place to calculate mass flow outside the optical field of a Gaussian beam, therefore we decided to link the c_1 heat_ parameter with a hypothetical wider “heat-related Gaussian beam” reproducing the heat conduction from the hot spot toward its outside and the corresponding temperature distribution. The necessary function of the growth of the “heat-related Gaussian beam” radius was taken from the measurements by the AFM technique of real widths of craters. The exact functions of c_1_, c_1 heat_ and c_2_ coefficients on the laser power used in our simulations are given in Appendix B.

**Figure 6:**
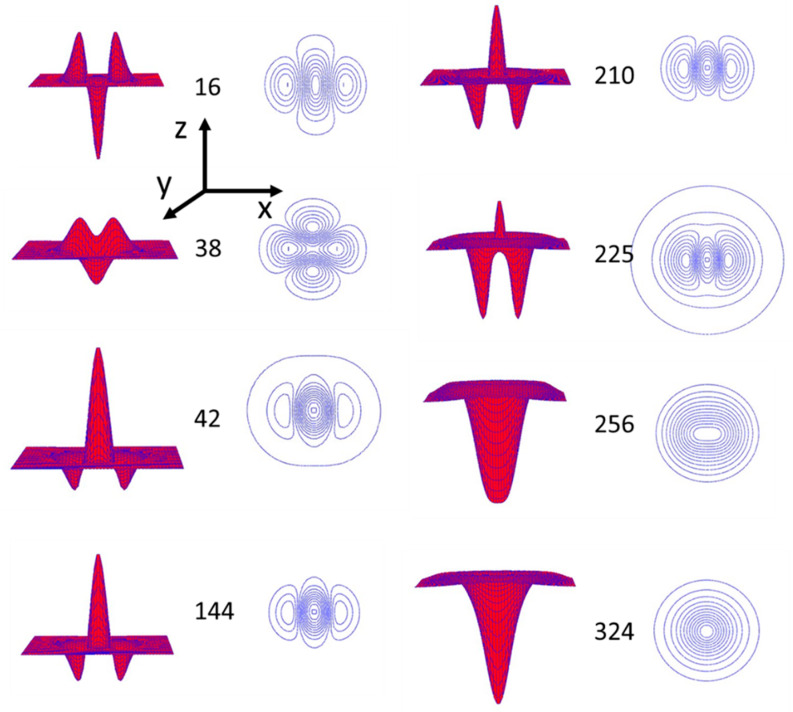
Exemplary 3D normalized projections of the surface deformations and maps of the mass redistribution induced in the azopolymer by the focused laser beam for three regimes of the used power: the weak power regime (0 to 36 a.u.); the first intermediate power regime (38 a.u.); the medium power regime (64 to 196 a.u.); the second intermediate power regime (210 to 225 a.u.); and the strong power regime (196 to 400 a.u.). Laser beam was set to be linearly polarized along the y-axis.

In Figure 6 we show simulations of the surface deformations across the three power regimes in 3D together with maps of the laser polarization-induced mass flows.

**Figure 7:**
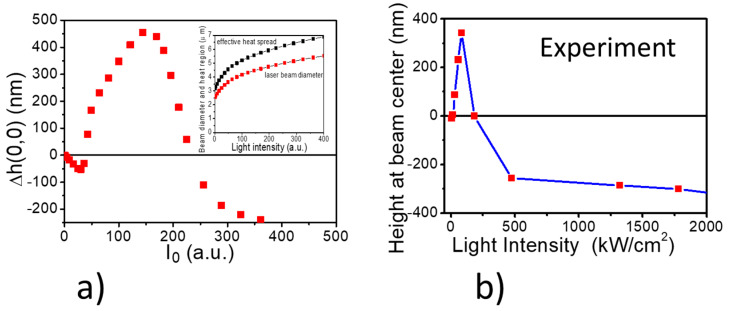
Plots or relief heights with respect to the polymer surface as obtained from (**a**) simulation and (**b**) the AFM profile measurements in the side-chain azobenzene poly(amide-imide). In an inset to Figure 7a we show the enlargement of the Gaussian profile for the thermal process of the mass flow versus apparent beam diameter, neglecting the heat spread.

Analysis of the simulation results presented in Figure 6 shows us that the azopolymer mass current is directed along the E-vector of the irradiating Gaussian beam and depends on the gradient of this field magnitude. This, together with the assumed incompressibility, is responsible for the formation of the side lobes or dips situated along the y-axis. This symmetry at the strong laser power is totally cancelled (see map at power of 324 a.u.) and the crater is surrounded by the circular rim. That means that the mass flow is driven mainly by the thermal effects. The transitions between the regimes are interesting and show an excellent agreement with the reliefs measured by AFM. Comparing the mass redistribution map for the weak power regime (16 a.u.) with that for the strong power regime (324 a.u.) one can notice the significant change of its character from a highly anisotropic to isotropic (circular) one, which corresponds to the cancellation of the birefringence observed by POM at the weak power regime. Especially after the transition from the weak to the medium power regime the birefringence starts to decrease from the center towards the outside. The ordering of *trans* molecules is effective and could be frozen only at low temperatures. With the temperature rise, when the optofluidization takes place, both ordering and birefringence diminish. In the medium power regime when a build-up of a sombrero-like structure takes place, a rapid flow of the mass at the beam center does not lead to an increase of the birefringence because at the Gaussian beam center the gradient of an electric field is equal to zero. In our model we add thermal effects, allowing the enlargement of the original beam waist, and we describe it with the c_1 heat_ coefficient starting to rise from zero value at around 70 a.u. of the laser power. Its magnitude above 250 a.u. of the laser power dominates the other effects. In Figure 7a we present the results of the relief height at the beam center as simulated from the model and in Figure 7b the experimental values of the relief height measured from the AFM experiment. The model parameters were chosen with the aim to mimic the experimental relief height values.

### 3.3. Thermocapillary Marangoni Flows

The processes involved into the surface deformation under direct laser inscription at 473 nm are evidently related to the photoisomerization of the *trans* and the *cis* azobenzene pending groups. The formation of a large number of *cis*-forms of azobenzene occurs mostly on the illuminated polymer surface leading to an apparent viscosity reduction and fluidization. In the numerical calculation model presented in this work, all relaxation processes counteracting the mass migration as viscoelasticity and lowering of the surface tension by the temperature were neglected. So, in the simulation they could not be observed, but they are observed in a real experiment (see Figure 4a where we marked the features not appearing in the simulations as Marangoni-related flows). Thermocapillary or the Marangoni convection is the well-known phenomenon observed in all liquids [38]. The temperature gradient or concentration gradient that appears at the surface changes the surface tension and the flow of liquid close to the surface from the low surface tension region toward the higher one takes place. In the thermocapillary effect the temperature gradient ∇T is the driving force. For most of the organic liquids far from their boiling points, the surface tension σ(T) lowers linearly with increasing temperature, therefore the stream of liquid is directed from the hottest point at the liquid surface (e.g., position of the focus of the laser beam) towards the outer edge. In the azobenzene liquid crystal and in the dye-doped organic solvents studied recently by us, these flows were observed under optical microscope [47,48,49]. The Marangoni flows are quantitively described by the Marangoni number *Ma* that compares the rate at which the thermal energy is transported by the surface tension gradient-driven flow to the rate at which the thermal energy diffuses:

(1) $$Ma=-\left(\frac{\partial \sigma }{\partial T}\right)\cdot \frac{L\Delta T}{\eta \alpha }$$

where (*∂σ/∂T*) is the rate of surface tension *σ* change with temperature, *L* is the thickness of liquid layer, *η* is the polymer viscosity in a liquid state and *α* is the thermal diffusivity. When the thermal diffusion dominates and viscosity is large, *Ma* is small and there is no flow. However, when the polymer, due to action of light and heat, enters into its liquid phase, flow (convection) occurs which is driven by gradients in the surface tension, and this usually happens when *Ma* > 60. The duration of the light irradiance is only 50 ms in our experiment, but it is sufficent to raise the temperature of the suface layer above T_g_. The streams of the fluid start to flow outside the heat source (Gaussian beam diameter) and at some distance from the focal point they solidify. The remnants of the described process are seen by us during analysis of several profiles of the surface deformation by the AFM technique, but only at strong irradiation levels (cf. Figure 4a, shadowed area). At the positions of these anomalies, gradients of the electric field of light as well as gradient of the light intensity do not exist. The simulation of this effect will be our next goal, but now it is premature as the surface tension of the molted polymer and its temperature dependence have never been measured. However, a surface tension modulation can also be driven by an asymmetrical distribution of the *cis*-azobenzene across the irradiated area of the azobenzene polymer film. The solutocapillary Marangoni effect has been noticed by Kim et al. [50] in the azobenzene-containing polymer film. Analysis of the topography changes allowed them to discover dissimilar capillary velocities that were associated with different azobenzene isomer concentrations. Clever uses of Marangoni flows have been described in a recent paper by Kitamura et al. [51]. They studied an inkjet printing process over azopolymer layers. The uniform UV irradiation via the surface tension modification induced Marangoni flows that allowed them to observe significant amplification in the permanent surface deformation. This technique provides a novel robust tool for surface microfabrication. Laser-heating-directed Marangoni flow on a dye-doped poly(ethyl methacrylate) film was demonstrated by Elashnikov et al. [52], and some Marangoni effects are also mentioned in a recent review paper by Dattler et al. [53].

**Figure 8:**
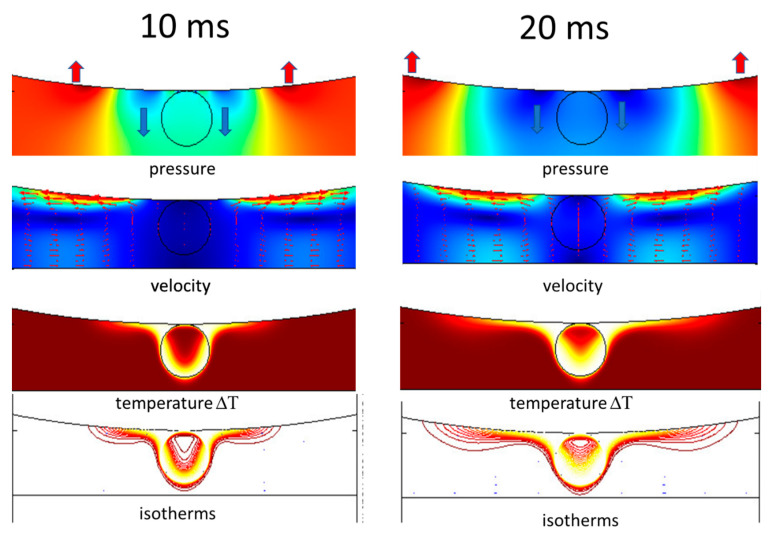
Schematic view of the processes taking place when an absorbing isotropic liquid is illuminated with a focused laser light (here represented as a circle of temperature 5 K above the glass transition temperature T_g_, with a radius of about 1.5 μm). The system has a cylindrical symmetry and here only cross sections of the pressure distribution, the velocity distribution of the mass flow, the temperature showing both diffusive as well as dominating convection flow and the isotherms are shown at 10 ms and 20 ms after launching the laser.

In order to better understand the thermocapillary Marangoni effect in the azopolymer we have performed preliminary simulations using COMSOL Multiphysics approach, the same as those presented in our previous papers [48,49,54]. Without the knowledge of the exact physical parameters of the melted liquid phase of the studied polymer, we performed simulations as for the typical organic liquid to show that the expected processes take place when the liquid phase is irradiated with the focused laser beam. We have assumed a 3-μm-thick layer of the melted liquid heated by a laser beam with its focal volume placed inside the layer (see the circles in Figure 8). 

In the simulation model the laser heating was equivalent to the excess temperature of the focal volume equal to 5 K above the T_g_ value and set on the circle. The calculations were performed up to 50 ms because irradiation time in our experiment lasted 50 ms. The results of simulations are shown in Figure 8 after 10 ms and 20 ms of laser action. Three processes that are visible from the modeling are: (i) the polymer flow close to the surface with increasing velocity from the warmer region towards the cooler one; (ii) the convection of heat accompanying the mass flow; and (iii) the pressure changes pushing up the polymer mass at some distance from the heat source.

In the top row of Figure 8, the red and blue arrows indicate the action of the pressure, with red representing the overpressure and blue the underpressure. That means that it is quite probable that at these points one can expect local surface deformation (bending). The positions of the overpressure change with the irradiation time and they can be frozen when the laser light is switched off (cf. shadowed area in Figure 4a). Therefore, the whole process can give quantitatively different responses (the remnants of the thermocapillary Marangoni effect) as they depend on the laser power, the light absorption, the liquid viscosity, and the amount of the *cis/trans* states. More adequate modeling adapted to an azopolymer irradiated with light is necessary to quantitively predict the surface deformation phenomena taking place when the laser power is able to melt the free upper layer of the polymer film.

## 4. Conclusions

In this work we performed a study on focused polarized laser light inscriptions in an azopolymer across irradiance intensity levels extending from ~0.4 kW/cm^2^ to ~400 kW/cm^2^. At weak light intensities (up to ~1.2 kW/cm^2^) the surface deformation is well described by existing theories involving dependence of polymer mass movement on electric field gradients. At medium and strong light intensities, thermal effects start to dominate the surface deformation. Assuming photofluidization of the polymer upper layer, one can expect the well-known thermocapillary Marangoni flows to occur which, up to now, have not been explicitly included in theories describing surface relief grating formation, but have been observed in other experiments. Our analysis involving POM observations, AFM topographical measurements and modeling based on advanced theories seems to prove that the Marangoni thermocapillary effect also plays an important role in surface deformation by direct laser writing. This effect is very sensitive to light exposure dose and does not show dependence on linear light polarization. We believe that this work is a preliminary step on the way to precisely evaluate Marangoni effects occurring in the whole family of azopolymers. The Marangoni flows diminish the lateral resolution of laser lithography but can be useful for more sophisticated shaping of the azopolymer surface.

## Figures and Tables

**Figure A1:**
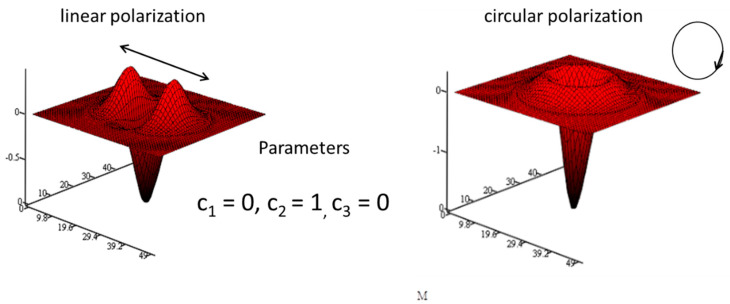
Reliefs in the azopolymer as a result of the irradiation with a Gaussian beam of the intensity I_0_ simulated on the basis of Equation (A1) with parameters *c*_1_ = 0, *c*_2_ = 1 and *c*_3_ = 0. Results are similar to that presented in a review paper [41].

## Appendix A

In Ambrosio et al.’s [11] model of the mass transport in the photochromic polymer films, exposed to the illumination of the light beam for a time t, the light-induced mass flow gives rise to a surface deformation Δ*h(x,y),* which follows from the solution of the Navier–Stokes equations under the assumption of the approximate polymer incompressibility. Δ*h(x,y)* has been calculated from Equation (A1):

(A1) $$\Delta h(x,y)=(c_{1}+c_{2})[\partial_{x}^{2}|E_{x}{|}^{2}+\partial_{y}^{2}|E_{y}{|}^{2}]+c_{1}(\partial_{x}^{2}|E_{y}{|}^{2}+\partial_{y}^{2}|E_{x}{|}^{2})+\;c_{2}\partial_{x}\partial_{y}(E_{y}^{*}E_{x}+E_{x}^{*}E_{y})+c_{3}(\partial_{x}^{2}|E_{z}{|}^{2}+\partial_{y}^{2}|E_{z}{|}^{2})+c_{S}[\partial_{x}Re(E_{z}^{*}E_{x})+\partial_{y}Re(E_{z}^{*}E_{y})]$$

Spatial electric field components *E_i_* are defined for a Gaussian beam including the *E_z_* light component appearing when the laser beam is tightly focused. In this work, we performed simulations of surface reliefs for a linearly polarized Gaussian beam of the form:

(A2) $$I\left(x,y,z\right)=I_{0}\cdot {\left(\frac{w_{0}}{w\left(z\right)}\right)}^{2}\cdot e^{\frac{-2\left(-x^{2}+y^{2}\right)}{{\left(w\left(z\right)\right)}^{2}}}$$

where w_0_ is the beam waist radius and the polarization has been set by defining *E_x_* = 0 and *E_y_* ≠ 0.

The parameters *c*_1_, *c*_2_, *c_3_* and *c_s_* of Equation (A1) are material dependent. They are specific for the microscopic molecular diffusion process characteristic for the used polymer, the azo-chromophore and the type of its linkage to the main chain. The coefficient *c*_1_ controls a mass transport current along the gradient of the light intensity regardless of the polarization, while *c*_2_ corresponds to the additional mass transport effect occurring when the light gradient is parallel to the optical electric field, and *c*_3_ gives a mass transport controlled by the intensity of the longitudinal component of the electric field. The exact meaning of these parameters is discussed in the publication quoted above [11]. However, the best way to determine the values for the studied azopolymer is a direct comparison of experimentally measured reliefs with the simulated ones and such adjustment of these parameters to achieve the exact shape and magnitude of the reliefs. As a test we simulated reliefs for the linearly and circularly polarized Gaussian beam, obtaining classically predicted and observed results (cf. Figure A1) which validated the method.

## Appendix B

In simulations of surface deformation under the tightly focused laser beam we have assumed functional dependencies of the key coefficients *c_i_* of the theory. They are described in function of the laser intensity at the central point of a Gaussian beam *I*(0,0) = *I*_0_ given in arbitrary units (a.u.) in the range 0 to 400 a.u. The parameter c_1_(*I*_0_) describes the response to light intensity gradients:

(A3) $$c_{1}\left(I_{0}\right)=c_{1max}-\left[\frac{c_{1min}}{\left(1+\left(\exp\left(-\frac{\left(I_{0}-I_{0trans\;1}\right)}{\tau_{1}}\right)\right)\right)}\right]$$

with *c_1max_* = −0.15, *c_1min_* = −0.2. The parameter *I_0trans 1_* = 200 a.u. describes at which laser intensity the function changes its sign from negative to positive and the parameter *τ*_1_ = 30 a.u. describes how wide the transition is. The evolution of the *c*_1_(*I*_0_) parameter is shown in Figure 5a by the black line.

The parameter *c*_2_(*I*_0_), in our simulation, has two components which behave differently at the different light intensities. The first component is responsible for directional mass movement along the y-axis i.e., along the electric field gradient and the second was added to express the destructive influence of the elevated temperature on the directed mass flow along the y-axis.

(A4) $$c_{2}\left(I_{0}\right)=c_{2max}-\left[\frac{c_{2min}}{\left(1+\left(\exp\left(-\frac{\left(I_{0}-I_{0trans\;2}\right)}{\tau_{2}}\right)\right)\right)}\right]+c_{22max}-\;\left[\frac{c_{22min}}{\left(1+\left(\exp\left(-\frac{\left(I_{0}-I_{0trans\;22}\right)}{\tau_{22}}\right)\right)\right)}\right]+3$$

with *c_2max_* = −1.0, *c_2min_* = 2.0, *I_0trans 2_* = 40 a.u. describing at which intensity the function exhibits a sign reversal and *τ*_2_= 3 a.u. describing how abruptly it occurs. For the second term of the Equation (B2) the parameters are the following: *c_22max_* = −1.0, *c_22min_* = −1.0, *I_0trans 22_* = 200 a.u. and *τ_22_*= 20 a.u. The evolution of the c_2_(*I*_0_) parameter is shown in Figure 5a by the red line. The parameters c_3_ and c_s_ are less important in our simulation and can be set to 0, though in some cases they cannot be neglected.

(A5) $$c_{3}\left(I_{0}\right)=0\;\mathrm{and}\;c_{s}\left(I_{0}\right)=0$$

The parameter *c_1_^heat^*(*I*_0_) is related to the action of heat released during irradiation with the strong light intensities and in simulation it is added to *c*_1_(*I*_0_). Its role is connected with the mass flow at the regions where all fields, i.e., the electric field of light and consequently its intensity, are negligible. We introduced it to describe the widening of the deformed area assuming an isotropic heat conduction process and respective thermal fluidization. This process came into play at relatively strong intensities and is dominating above 200 a.u. as shown in Figure 5a by the green line. The functional dependence of the parameter *c_1_^heat^*(*I*_0_) was assumed as:

(A6) $$c_{1}^{heat}\left(I_{0}\right)=c_{1max}^{heat}-\left[\frac{c_{1min}^{heat}}{1+\left(\exp\left(-\frac{\left(I_{0}-I_{0trans}^{heat}\right)}{\tau_{1heat}}\right)\right)}\right]$$

with $c_{1max}^{heat}$ = 0, $c_{1min}^{heat}$ = −1.5,$\;I_{0trans}^{heat}$ = 180 a.u. and *τ_1heat_* = 30 a.u.
